# The Adoption of Roles by Primary Care Providers during Implementation of the New Chronic Disease Guidelines in Urban Mongolia: A Qualitative Study

**DOI:** 10.3390/ijerph13040407

**Published:** 2016-04-07

**Authors:** Oyun Chimeddamba, Darshini Ayton, Nansalmaa Bazarragchaa, Bayarsaikhan Dorjsuren, Anna Peeters, Catherine Joyce

**Affiliations:** 1 School of Public Health and Preventive Medicine, Monash University, Melbourne 3004, Australia; Darshini.Ayton@monash.edu (D.A.); catherine.joyce@monash.edu (C.J.); 2 Mongolian Association of Family Medicine Specialists, Ulaanbaatar 14210, Mongolia; 3 Mongolian Public Health Professionals’ Association, Ulaanbaatar 210646A, Mongolia; nansaab@hotmail.com; 4 Department for Health Systems Governance and Finance, World Health Organization, Geneva 1202, Switzerland; bayarsaikhand@who.int; 5 School of Health and Social Development, Deakin University, Geelong 3220, Australia; anna.peeters@deakin.edu.au

**Keywords:** clinical guidelines, family health centre, primary care, role delineation, Mongolia

## Abstract

(1) *Background*: In 2011, new chronic disease guidelines were introduced across Mongolia. No formal advice was provided regarding role delineation. This study aimed to analyse the roles that different primary care providers adopted, and the variations in these, in the implementation of the guidelines in urban Mongolia; (2) *Methods*: Ten group interviews with nurses and ten individual interviews each with practice doctors and practice directors were conducted. Data was analysed using a thematic approach based on the identified themes relevant to role delineation; (3) *Results*: There was some variability and flexibility in role delineation. Factors involving teamwork, task rotation and practice flexibility facilitated well the guideline implementation. However, factors including expectations and decision making, nursing shortage, and training gaps adversely influenced in the roles and responsibilities. Some role confusion and dissatisfaction was identified, often associated with a lack of training or staff turnover; (4) *Conclusions*: Findings suggest that adequate ongoing training is required to maximize the range of roles particular provider types, especially primary care nurses, are competent to perform. Ensuring that role delineation is specified in guidelines could remove confusion and enhance implementation of such guidelines.

## 1. Introduction

Hypertension and diabetes are key risk factors for cardiovascular disease (CVD) and, since 2000, CVD has been one of the leading causes of mortality in Mongolia [1,2]. In 2013, the prevalence of high blood pressure was 27.5% and diabetes 6.9% [3]. In 2011, a working group of the Ministry of Health (MOH) Mongolia in co-operation with Millennium Challenge Account-Mongolia (MCA-M) Health Project developed and published clinical guidelines on arterial hypertension and diabetes (hereafter referred to as the guidelines) [4]. The guidelines aimed to improve the population wide prevention of hypertension and diabetes and reduce the overall CVD morbidity and mortality through early detection of associated risk factors. The content and recommendations of the guidelines were based on recent international guidelines on the management of hypertension and diabetes such as the World Health Organization (WHO) and the European and Finnish national guidelines. These guidelines are the first public health oriented and primary care driven evidence-based practice recommendations for the prevention, diagnosis and management of hypertension and diabetes to assist Family Health Centres (FHCs) in their work. The guidelines were primarily relevant for FHCs and covered a broad range of screening and management aspects of hypertension and diabetes, including taking body measurements, testing blood sugar and cholesterol, assessing risk scores and providing advice regarding lifestyle modification interventions.

Primary care in Mongolia operates predominantly through state funded private FHCs, which provide universal access to health care for individuals, families and communities [5]. Role delineation refers to a description of the responsibilities, functions and activities of a health care professional in a specific role [6]. The existing model of family practice environment in Mongolia is broadly based on a model of multidisciplinary team health care delivery where collaborative practice is undertaken to provide health care [7,8]. In this model of practice each health professional contributes to patient care and increase the likelihood of guideline adoption although with some overlapping scope of practice [9].

In Mongolian FHCs, a practice doctor maintains primary clinical decision making authority and the largest scope of practice with delegation of care to other types of providers such as primary care nurses and social workers [10]. Practice directors are commonly those with a medical background, and they serve as team leaders and delegate the entire clinical practice to family doctors and nurses. Legally, overall dimensions of roles and responsibilities for practice doctors and primary care nurses are regulated under professional standards and nationally agreed model job descriptions which were approved by Ministerial Orders in 2012 [11,12]. The Ministerial Orders define the minimum requirements for each professional role and also determine the key required performance standards of staff employed within facilities.

The implementation of clinical guidelines and their relationship to role delineation is not well represented in research literature [13,14,15]. One reason for this gap may be that clinical guidelines themselves often fail to nominate the service provider primarily responsible for implementing recommendations. This is the case with the guidelines in Mongolia as they do not formally provide advice regarding role delineation for primary care providers. There was an implicit expectation that the guidelines would be largely delivered based on the existing model of family practice. However, the guidelines had an increased emphasis on prevention of chronic diseases, and as such represented an expansion in the scope of work of FHCs. In particular, there was an opportunity to maximize the use of nurses’ skills in delivering preventive health care. We were interested in whether the guidelines provided an impetus to potentially reconsider the roles and responsibilities of various members of the primary care team and how these new areas of work were implemented across members of the teams within each FHC. This is an important aspect of the overall process which facilitates the translation of best practice guidelines into clinical practice. It may be that prior specification of roles and responsibilities within the guidelines improves the effectiveness of their implementation. Therefore, in this study we aimed to examine the characteristics of primary care providers’ role delineation in the context of the guideline implementation.

## 2. Materials and Methods

A qualitative design was employed. This study was a part of the main qualitative research published recently which explored the barriers and enablers to the implementation of the guidelines [16]. The current study examines the influence of role delineation in the implementation of the guideline recommendations to understand how these recommendation were delivered by different primary care providers within each FHC. The methods for this research have been described in detail elsewhere [16].

In brief, the research was conducted in Ulaanbaatar City, Mongolia. An initial sampling frame was a list of all the FHCs (*n* = 136) located in Ulaanbaatar City from the public domain of the Mongolian Association of Family Medicine Specialists. Qualitative studies often use purposive sampling methods, which are based on theoretically relevant characteristics that guide selection of participants. However, in our study, we used random sampling to select ten FHCs from the sampling frame to ensure a variety of diverse views were obtained. After selection the characteristics (location, practice size and staffing) of the FHCs were examined to ensure the practices were representative. We randomly chose ten practices using a sequence of random numbers. At each practice, individual interviews were conducted with the director, the practice doctor and the nurse. This generated approximately 30 interview transcripts in total. However the researcher was flexible in relation to the total number of FHCs recruited to the study being dependent on reaching data saturation (the point at which no new information emerged). Data saturation was reached after interviews with the staff at the seventh FHC. However, data collection at the remaining three practices was completed for a purpose of confirmation and verification of the study findings. We considered this an adequate sample as per qualitative research methods [17]. Once ten FHCs had been randomly selected, the researcher contacted heads of the selected FHCs explaining the purpose of the research and asked permission for their staff to take part in the study. After agreement was made with the heads of the selected FHCs, primary care providers (PCPs) were invited to participate in the study. PCPs interested in participating were asked to contact the researcher directly. They were informed that participation is voluntary and their identity will be kept strictly confidential. In addition, PCPs were aware that they have a full right to withdraw if they decide to do so. All participants signed informed consent. Ethical approvals for the study were obtained from the Monash University Human Research Ethics Committee (CF13/3265-2013001701) and the Ministry of Health (Mongolia) Ethics Committee (application#4 of 6 Dec, 2013).

Summary description of the participating FHCs was as follows. The majority of the FHCs were established around 2000 and offered primary care services to 6000–15,800 (4 FHCs: 6000–8000; 3 FHCs: 8000–10,000; 3 FHCs: 10,000–15,800) residents living in the given catchment areas. They were located in community settings to make them accessible to households in their catchment areas. Typically FHCs consisted of three to four family doctors on average with a nurse assigned to each doctor. Most of the personnel were quite experienced and working at FHCs for more than 10 years. Most FHCs were open during regular office hours with some additional hours at weekends. The mission of the FHCs was to enhance the quality and accessibility of public health services and to promote healthy behaviors through health education and health promotion. Table 1 provides an overview of the PCPs interviewed in this study.

A total of 40 PCPs from ten general practices in urban Mongolia participated in the study. Qualitative research does not seek to be generalizable, rather participants were recruited to provide insight into the phenomena of guideline implementation. Research by Guest *et al.* (2006) found that data saturation—the point where no new information or themes occur in the data—is achieved at 12 participants [18]. Therefore an overall sample of 40 was deemed to be adequate. Data collection took place between November 2013 and February 2014. Ten practice directors and ten practice doctors participated in semi-structured individual interviews. Twenty primary care nurses participated in ten group interviews. All interviews were audio-recorded, transcribed verbatim, translated into English and imported in NVivo 10 for Windows (QSR International, Melbourne, Australia) for data management and analysis [19]. Researcher field notes were retained. Oyun Chimeddamba, native Mongolian, who conducted the interviews in the local language, read the transcripts two to three times, and developed a coding guide using a process of deductive coding based on the interview schedule. The first round of coding involved the development of these deductive codes. The second round of coding was inductive, and Oyun Chimeddamba identified and confirmed emerging themes through open, axial and thematic coding [20,21]. Transcripts and emerging themes were circulated to Anna Peeters and Darshini Ayton and discussed for verification. Two key emerging themes were identified relevant to the role delineation of providers from analysis of transcripts: (1) factors that influenced role delineation and (2) characteristics of role delineation perceived to be associated with effective guideline implementation. Each key theme contained a number of subthemes which supported and reinforced the context of the themes. Table 2 provides an overview of the two themes and corresponding sub-themes. Subthemes included expectations and decision making, training attendance and nurse shortages/turnover, reliance on practice doctors were identified as influencing factors for role delineation. Subthemes such as teamwork, task rotation, and practice flexibility were seen as positive drivers in role delineation that supported the guideline implementation. 

## 3. Results

To provide context of the different roles in the FHCs, an overview of each of the provider roles as described by the participants is detailed below.

### 3.1. Description of the Roles of PCPs

Most nurses described their roles and responsibilities in implementing the guidelines as extensive. In most of the FHCs nurses usually performed body measurements, checked blood pressure, took blood samples for glucose and cholesterol tests and provided general lifestyle recommendations.

“…My role as a nurse involves detection of early signs of developing chronic diseases and health education designed for the general public. I try to encourage even one patient drinking a salty tea to switch to a less or salt free tea”.

(Nurse, FHC3)

In general, practice doctors examined risk factor assessment for hypertension and diabetes, suggested specific lifestyle recommendations, made appropriate clinical decisions and monitored cases for follow up based on the results of the tests performed by nurses. Practice doctors were involved in making referral decisions for necessary clinical cases.

“…I check the risk assessment chart and blood test results of patients for hypertension and diabetes, diagnosing diseases, and make clinical decisions whether to send a patient to a district health centre for further examination or monitor a suspected case at FHC”.

(Practice doctors, FHC9)

FHC practice directors commonly perceived themselves as being in charge of informing their personnel of policy releases, managing the guidelines implementation, leading the team approach to primary care delivery and monitoring staff performance.

“…I am responsible for bridging my staff with specialists at the district health centre, initiating action plans to implement health programs and projects, steering local health campaigns, and monitoring the quality of staff performance”.

(Practice director, FHC2)

### 3.2. Factors that Influenced Role Delineation

Factors identified as influencing role delineation in the adoption of the guidelines included expectations and decision making, attendance at training on the implementation of guidelines and nurse shortages/turnover, reliance on practice doctors.

#### 3.2.1. Expectations and Decision Making

In general, primary care providers felt that they were expected to deliver the guideline recommendations based on the existing model of general practice. In this respect, the providers generally were clear within their own practices about their respective roles. However, these roles differed between practices. For instance, a few of the interviews with nurses revealed that they had no clear understanding of what the formal role boundaries were despite being aware of the guideline arrival into practice. As a consequence, a small number of nurses in particular felt that their role was limited to only assisting and supporting the doctors.

“…In most instances, I assume that my role is suggesting general advice on the lifestyle modification interventions. Hmm, but I am not heavily involved in the guidelines because I am not told what to do. Thus, I just try to help our doctors”.

(Nurse, FHC3)

#### 3.2.2. Attending Training on the Implementation of the Guidelines

In general, providers who reported having attended training perceived themselves as the key implementers of the guidelines and therefore felt they had more ownership than others over the roles and responsibilities. By contrast, several nurses who did not attend any training courses indicated that they had not been formally granted a particular role in the guideline implementation.

“…Despite having had no formal training, I am aware of the guidelines. I have been less involved in their ongoing implementation because I have no clear understanding of the exact nature of the role. The guidelines do not set out tasks to complete and our managers have not allocated these to us in a consistent manner”.

(Nurse, FHC7)

#### 3.2.3. Nurse Shortages/Turnover and Reliance on Practice Doctors

Turnover also appeared to be a barrier to consistent implementation of guidelines. A few practice directors reported that sometimes nurses who had attended training courses related to the guidelines left their jobs, and the other nurses were not able to take over the role as they had not attended the relevant training. Therefore, due to shortage of trained nurses at FHCs, practice doctors were increasingly becoming responsible for performing most tasks associated with the guidelines in addition to making clinical decisions.

“…I did not attend any training on the NCD prevention, thus I am not much involved in the process of the guidelines. Only one nurse attended that, but she has left her job. Since then, our doctors became involved in taking blood tests for sugar and cholesterol”.

(Nurse, FHC5)

### 3.3. Characteristics of Role Delineation Perceived to be Associated with Effective Guideline Implementation

Participants in this study identified a number of factors that they believed led to more effective guideline implementation in relation to role delineation. In particular, teamwork and task rotation—the continuous weekly changes of tasks amongst providers—and practice flexibility were seen as positive drivers in role delineation that supported the guideline implementation.

#### 3.3.1. Teamwork

A teamwork approach was valued by providers, who expressed enthusiasm for the shared responsibilities and functions, including the current activities common to the roles. The data indicated that leadership at various levels was critical for successful implementation. Practice directors and practice doctors pointed out that they viewed their support for the guidelines as essential, as they perceived themselves as being central to providing guidance and legitimacy for the guideline delivery.

“…The implementation of the guidelines depended on us. Doctors and nurses should participate equally in implementing the guidelines because it requires teamwork and ongoing effort”.

(Practice director, FHC1)

#### 3.3.2. Task Rotation

There were a small number of FHCs where task rotating over a week or two weeks was initiated under the leadership of practice directors. For example the tasks of practice doctors and nurses would be rotated to different practice doctors and nurses either weekly or fortnightly. The directors believed the process of task rotation improved the clinical performance and professional competency of their staff.

“…The roles and responsibilities of my employees are the same because every doctor and nurse rotates their positions on a weekly basis. It helps to work as a team and substitute other’s position. Basically everyone has to work in diverse profiles by delivering a wide range of care and services”.

(Practice director, FHC10)

#### 3.3.3. Practice Flexibility

Practice flexibility refers to working in a dynamic environment, which was perceived to be beneficial to personnel at FHCs and patients visiting FHCs [22,23]. For instance, numerous practice doctors revealed that when they were overloaded with emergency or acute clinical cases at FHCs, nurses commonly became more involved in the detection and screening of risk factors for hypertension and diabetes normally delivered by practice doctors. Similarly, some nurses acknowledged that if they were coping with higher demands, such as dealing with vaccination, home visits for newborn babies and seasonal outbreaks of influenza, practice doctors frequently came on board to perform preventative tasks that would usually have been carried out by nurses.

“…We, doctors examine patients, assess risk appraisal chart for diabetes and hypertension. But, when we are too busy, nurses get more involved in the risk assessment, BMI calculation and lifestyle interventions. We work together. We are not isolated from each other, the roles are not divided totally”.

(Practice doctor, FHC7)

## 4. Discussion

The present study is the first to explore the factors that either facilitated or adversely influenced the role delineation of different primary care providers in response to the hypertension and diabetes guideline implementation at the primary care level in urban Mongolia. Although no roles were specified in the guidelines, the results demonstrate that many similarities in role delineation were observed across FHCs in urban Mongolia. The majority of FHCs in this study implemented the guideline recommendations according to the existing model of family practice, which was characterised by team-based care composed of groups of health care professionals, including practice doctors and primary care nurses [8]. Notably, the existing model of practice clearly played a major role in the delivery of the guideline interventions in terms of role delineation of providers. However, a few nurses related that they were granted a secondary role with lesser responsibilities in terms of tasks and clinical decision making processes if compared to the practice doctors. They pointed out their role was confined to assisting the practice doctors. This may underestimate the capacity of nurses to play an essential role in the prevention and early detection of hypertension and diabetes in various clinical settings [24,25]. Guidelines deliver clear evidence that public health and clinical care requires a multidisciplinary approach that includes a range of specialised and generalist practitioners, nurses, and allied health professionals, where appropriate [13,22]. In addition, evidence suggests that nurses make valuable contributions in multidisciplinary teams to address the burden of noncommunicable diseases (NCDs) and reduce risk factors by implementing practices, such as those outlined in the evidence-based clinical guidelines [26]. For example, the Nursing and Midwifery Program at WHO strongly acknowledged that many countries recognize the vital role of nurses in improving health service delivery, as they may be the only providers to deliver health services [25,27]. The 2012 WHO Global Forum for Government Chief Nursing and Midwifery Officers emphasized the need to support the role of nursing in NCD prevention and management and that these roles should be reflected in updated job descriptions and role delineations [28]. Therefore, given the existing challenges, the next iteration of the guidelines should carefully consider specifying roles and responsibilities of nurses in the delivery of guideline interventions so that nurses are recognized as key team members.

Despite being able to adopt the traditional method of practice to implement the guideline recommendations, this study identified a number of notable aspects that influenced role delineation which also impacted the guideline implementation. These included decision making, training attendance, nurse shortage, teamwork, task rotation, and practice flexibility. It was apparent that practice doctors mainly held the management or decision making role, having control of work and the authority to make decisions in pursuit of the guideline implementation. Nurses appeared to have less authority for decision-making and this finding was in line with a number of official jurisdictions in Mongolia and elsewhere [12,29,30]. Research in the US and Canada highlighted the importance of nurse involvement at all stages in the management of hypertension and diabetes, from prevention to treatment, so that physicians are able to focus on unstable and complex patients [31,32,33]. Based on the evidence of successful clinical decisions mainly for chronic diseases made by nurses, we suggest that task shifting, where appropriate, to less specialized health workers such as nurses would be possible in the management of NCDs. This process of delegation can be viewed as a potential solution to improve the efficiency of FHCs.

Training participation was identified as a significant aspect that influenced role delineation in response to the guideline implementation. Trained providers indicated that they were more involved than their untrained counterparts in the process and showed their full participation in a wide range of interventions recommended in the guidelines. In certain cases, practice nurses who did not attend training felt limited in their ability to perform roles and responsibilities associated with guideline implementation. Some nurses were not given the same opportunity as practice doctors to attend training. The observed results in this study are supported by other research findings suggesting that proper training for nurses fosters high quality care and enhances expertise in the coordination and integration of such care [33,34]. Therefore, ongoing training opportunities should be made readily available for FHC personnel, especially nurses.

The findings in this study indicate that teamwork was perceived to be important for guideline implementation. Effective cross-professional collaboration and increased knowledge and skills of team members gained through ongoing training opportunities lead to continued improvement in the quality of care [8,35,36]. For example, the findings of the meta-analysis by Carter *et al.* in 2012 demonstrated that teamwork is effective in managing hypertension as it provides timely and collaborative care [35]. Moreover, Katon *et al.* found that quality of patient care can be improved by rearranging the roles and responsibilities of primary care physicians, nurses, and allied health professionals as a team in more coordinated fashion [31]. Hence, we suggest that future evidence-based recommendations should set up clear strategies on teamwork.

Flexibility in the workplace may lead to greater skill development and serve to motivate primary care providers and reduce frustration for them. Role flexibility and rotation enhanced delivery of the guideline recommendations and led to primary care providers being able to adopt diverse roles depending on the context and circumstances of the practice.

Our study is limited by the relatively small number of PCPs in the sample and the fact that they were recruited from urban areas of Mongolia. Despite this, the roles and responsibilities adopted by all types of PCPs were relatively consistent across the ten FHCs. However, the present study could have been strengthened by recruiting the similar providers from rural locations, for the obvious reason that their perceptions would provide an interesting comparison with our findings.

## 5. Conclusions

Our study has investigated the roles and responsibilities PCPs have adopted and variations in these roles across FHCs in the implementation of the guidelines in urban Mongolia. Understanding the processes facilitating the translation of evidence-based guidelines into clinical practice, and investigation of the roles and responsibilities adopted by primary care providers for the implementation of the guidelines, is an important precondition for guideline implementation strategies. Although the roles and responsibilities adopted were primarily based on the existing model of practice, there were a number of factors that influenced role delineation in the delivery of the guideline recommendations. The factors including expectations and decision making, nursing shortages, and training gaps resulted in frustration, being less responsible and insufficient skill development. Equally teamwork, task rotation and practice flexibility appeared to positively facilitate effective role delineation across all types of providers. Therefore, adequate ongoing training is required to maximize the range of roles particular provider types, especially primary care nurses are competent to perform. Ensuring that role delineation is specified in guidelines can remove confusion or disquiet so that there is better coherence and understanding of roles across all the different providers.

## Figures and Tables

**Table 1:** Study participants.

Group of People Involved	Number and Percentage of People Interviewed	Number of Interviews Conducted	Type of Interviews
Practice directors	10 (25.0%)	10	Individual interviews
Practice doctors	10 (25.0%)	10	Individual interviews
Primary care nurses	20 (50.0%)	10	Group interviews
Total	40 (100.0%)	30	

**Table 2:** Identified themes and sub-themes.

Themes	Factors That Influenced Role Delineation	Characteristics of Role Delineation Perceived to be Associated with Effective Guideline Implementation
	Expectations and decision making	Teamwork
Sub-themes	Attendance at training on the implementation of the guidelines	Task circulation/task rotation
	Shortage of nurses/turnover and reliance on practice doctors	Practice flexibility

## Abbreviations

The following abbreviations are used in this manuscript:

Guidelines	Clinical guidelines on arterial hypertension and diabetes
FHC	Family health centre
MCA-M	Millennium Challenge Account-Mongolia
MOH	Ministry of Health
NCD	Noncommunicable disease
PCP	Primary care provider
